# Supplementary material for: Remote homology search with hidden Potts models
Source: PLoS Comput Biol. 2020 Nov 30;16(11):e1008085. doi: 10.1371/journal.pcbi.1008085 (PMC7728182; doi:10.1371/journal.pcbi.1008085)
Supplement: Attachment — Submitted filename: Reproducible_report_PCOMPBIOL_D_20_01035.pdf [file pcbi.1008085.s003.pdf]

CENTER FOR REPRODUCIBLE BIOMEDICAL MODELING

Director: Professor Herbert M. Sauro
University of Washington, Seattle, WA
https://reproduciblebiomodels.org

Reproducibility report for: Remote homology search with hidden Potts models.
Submitted to: PLoS Computational Biology
Manuscript number/identifier: PCOMPBIOL-D-20-01035

Curation outcome summary: Were able to use the provided scripts to obtain, build, and install the software presented in this submission. Results from the provided benchmark problem could be reproduced.

Box 1: Criteria for repeatability and reproducibility

- [x] **Model source code provided:**
  - [x] Source code: a standard procedural language is used (e.g. MATLAB, Python, C)
    - [x] There are details/documentation on how the source code was compiled
    - [ ] There are details on how to run the code in the provided documentation
    - [ ] The initial conditions are provided for each of the simulations
    - [ ] Details for creating reported graphical results from the simulation results
  - [ ] Source code: a declarative language is used (e.g. SBML, CellML, NeuroML)
    - [ ] The algorithms used are defined or cited in previous articles
    - [ ] The algorithm parameters are defined
    - [ ] Post-processing of the results are described in sufficient detail
- [ ] **Executable model provided:**
  - [ ] The model is executable without source (e.g. desktop application, compiled code, online service)
    - [ ] There are sufficient details to repeat the required simulation experiments
- [x] **The model is described mathematically in the article(s):**
  - [x] Equations representing the biological system
  - [ ] There are tables or lists of parameter values
  - [ ] There are tables or lists of initial conditions
  - [ ] Machine-readable tables of parameter values
  - [ ] Machine-readable tables of initial conditions
- [ ] **The simulation experiments using the model are described mathematically in the article:**
  - [ ] Integration algorithms used are defined
  - [ ] Stochastic algorithms used are defined
  - [ ] Random number generator algorithms used are defined
  - [ ] Parameter fitting algorithms are defined
  - [ ] The paper indicates how the algorithms yield the desired output

Box 2: Criteria for accessibility

- [x] Model/source code is available at a public repository or researcher's web site
    - [ ] Prohibitive license provided
    - [ ] Permissive license provided
    - [x] Open-source license provided
- [ ] All initial conditions and parameters are provided
- [ ] All simulation experiments are fully defined (events listed, collection times and measurements specified, algorithms provided, simulator specified, etc.)

Box 3: Rules for Credible practice of Modeling and Simulation[a]

[a]Model credibility is assessed using the Interagency Modeling and Ananlysis Group conformance rubric: https://www.imagwiki.nibib.nih.gov/content/10-simple-rules-conformance-rubric

- [x] Define context clearly: Extensive
- [x] Use appropriate data: Extensive
- [x] Evaluate within context: Extensive
- [ ] List limitations explicitly: Insufficient
- [x] Use version control: Extensive
- [x] Document adequately: Extensive
- [ ] Conform to standards: Insufficient

Box 4: Evaluation

- [x] Model and its simulations could be repeated using provided declarative or procedural code
- [ ] Model and its simulations could be reproduced

Summary comments: Supplemental material for this submission included bash scripts which we used to successfully obtain, build, and install the software described in this manuscript. We used the tutorial supplied with the manuscript to successfully recreate results from the Twister ribozyme benchmark dataset. No documentation is provided describing how to use these tools to reproduce other results presented in the manuscript. The computational requirements of performing these simulations make it difficult to repeat them, having the well documented benchmark problem provides confidence that with further documentation all simulation results presented in this manuscript could be reproduced.

Anand K. Rampadarath[1], PhD
Curator
Center for Reproducible Biomedical Modeling

David P. Nickerson, PhD
Curation Service Director
Center for Reproducible Biomedical Modeling

Auckland Bioengineering Institute,
University of Auckland

[1] Email: a.rampadarath@auckland.ac.nz
