# Supplementary material for: Remote homology search with hidden Potts models
Source: PLoS Comput Biol. 2020 Nov 30;16(11):e1008085. doi: 10.1371/journal.pcbi.1008085 (PMC7728182; doi:10.1371/journal.pcbi.1008085)
Supplement: Attachment — Submitted filename: reviewer_response_WilburnEddy20.pdf [file pcbi.1008085.s004.pdf]

Howard Hughes Medical Institute

9 October 2020

Editors, PLOS Computational Biology

Dear editors,

We thank the referees for helpful and constructive comments on our manuscript “Remote homology search with hidden Potts models” (PCOMPBIOL-D-20-01035), and we thank you for the opportunity to submit a revised manuscript. Below we summarize each reviewer’s main points and reproduce each of their specific points, with our responses and revisions. We renumbered each specific point for ease of cross-referencing.

Reviewer #1:

Reviewer #1 describes the work as “generally well executed”, that “its conclusions convinced me”. The reviewer describes two weaknesses. First, they say the “experiments use only 3 different RNAs, making them arguably somewhat anecdotal,” but “I can't credibly argue that the overall conclusions… are likely to change with more RNAs.” Second, the reviewer says, “the paper is arguably a kind of negative result… HPM(s) don't work that well”, but the basic approach is a reasonable one that was deserving of investigation, and the paper gives important information”. We agree with these comments. Other comments were described as minor, which we address as follows:

> (1) W.r.t. this sentence: "A Potts model expresses the probability of a homologous sequence as a function of primary conservation and all possible pairwise correlations between all consensus sites in a biological sequence (i.e., consensus columns in a multiple sequence alignment)." : for readers not in the field, it might be good to define the concept of consensus columns explicitly. It's kind of hinted at, but never defined.

The reviewer is that we did not explicitly define consensus columns. We define consensus columns to be those in a multiple sequence alignment (MSA) with fewer than 50% gaps. We revised in the Results section to include this explicit definition.

> (2) The journal for ref 50 is missing. I see that this is a preprint, but there needs to be more information on where it is.

Thank you, we corrected this citation.

> (3) I found the notation P_m(x_m) a bit strange (e.g., in equation 2). I think this is the probability of

Sean R. Eddy
Investigator

Ellmore C. Patterson Professor
Molecular & Cellular Biology, and Applied Mathematics
Harvard University, Biological Laboratories 1008A
16 Divinity Avenue, Cambridge, Massachusetts 02138

+1-617-496-6757 | seaneddy@fas.harvard.edu | **eddylab.org**

x_m according to the model 'm'. Wouldn't it be more conventional to write this as P(x_m|M), and state that M is the model?).

Lowercase "m" in this case refers to "match" rather than "model." $P_m(\vec{x}_m)$ is the probability that the match states in the hidden Potts model generate match sequence $\vec{x}_m$. We made revisions to clarify this notation. In the Results section, we have added a clarifying statement that lowercase "m" refers to "match", lowercase "i" to "insert", and lowercase "t" to "transition". In addition, we have changed the notation of the match sequence probability under an HPM from $P_m(\vec{x}_m)$ to $P_{Potts}(\vec{x}_m)$ to increase clarity.

(4) I don't get why the model is broken into sub-models (as I understand it), e.g., p_t(\rho)

Under an HPM, $\vec{x}_m$ corresponds to the characters emitted by the model's match states; $\vec{x}_i$ to the characters emitted by the insert states; and $\vec{\sigma}$ (formerly $\vec{\rho}$, see below) to the state path through the model. Each of these variables is generated differently from a distinct probability distribution: $\vec{\sigma}$ via a Markov chain between states; $\vec{x}_i$ from the site-independent emission probabilities of the insert states in $\vec{\sigma}$; and $\vec{x}_m$ from the Potts distribution. We have added a sentence to explain this in the text.

(5) In equations (5) and (6), lower-case 'p' occurs in the right-hand side of the equation. I assume these should be a capital 'P', e.g. the probability of x_i_j in equation (5)? Otherwise, I'm not clear on what they represent.

We agree this notation is confusing. We now use capital P in these equations.

(6) In equation (6), the symbol \rho_n represents the states of the HPM. This could be explicitly stated. Secondly, I think it might help if the states were represented by s_n. In this equation the p and \rho look similar. Also, I got to this point in the paper believing that the path variable was \overrightarrow{p} and not \overrightarrow{\rho}. I think s would be more conventional.

To eliminate confusion between P and ρ, we now denote an HPM state path with $\vec{\sigma}$ rather than $\vec{\rho}$. This change maintains the tradition of using a Greek letter to denote a hidden state path, as is often done with profile hidden Markov models.

(7) I'm not sure I understand the description of \Lambda in equation (6). Does \rho include deletion characters or not? I think it does (since deletion characters must correspond to states), but the phrasing makes me wonder. Also, why is the variable L introduced here, when it's only actually used a few pages later? I found the latter clause in the sentence (page 7, line 165) more confusing than helpful. If it's left in, I'd recommend changing the semicolon to a new sentence (it looked like a comma when I first read it), and something like "Here, \Lambda is the total number of states in \rho. Given that \rho includes deletion characters, \Lambda is at least as large as the number of non-gap characters in x." But really, I think this should be moved later in the text.

$\vec{\sigma}$ (formerly $\vec{\rho}$) does not contain delete states. Instead, delete characters are emitted by match states in $\vec{\sigma}$. We have added an explanation to clarify this difference.

We have moved the introduction of L to the beginning of the "Hidden Potts models emit variable length sequences" subsection, where we first define unaligned sequence $\vec{x}$.

Additionally, we have removed the semicolon and clarified relationship between L, Λ, and $\vec{\sigma}$.

> (8) It would be nice to remind the reader of the meaning of M on page 10? Also, I think the variable L should be defined here. I believe this is the same as the L on page 7.

For clarity, we now remind the reader of the definitions of L and M at this point.

> (9) Since Potts models don't have a notion of insertions, the Potts model is presumably using every column in the input alignment (even gappy ones), right? In this case, is HMMER told to also treat all columns (even gappy ones) as consensus columns? I think this would be good to clarify.

We only train the Potts model on consensus columns, as defined by HMMER. We made revisions to explain this process more clearly in the "Training an HPM" subsection.

> (10) I have some questions about this sentence: "As Z is a constant for a particular model, scores can be compared relatively within a single database search with a given query HPM, but not qualitatively across different query HPMs (with different unknown Z's).". I get why searches with different HPM models (which have different implicit values of Z) can't be compared. But, this sentence implies that searches with the same query HPM of different databases are not comparable (because it specifies "within a single database"). Is this simply because the sizes of the databases are (in general) different, and so the statistical significance of a given score changes? Or is there some other reason?

There are two issues in play here: normalized probability distributions and statistical significance values. Regarding normalization, each HPM has a unique partition function value, Z. On the other hand, the partition function is independent of target sequence; each model has one and only one Z. Therefore, we can relatively rank log odds scores of target sequences that have been scored by the same query HPM.

However, knowing Z (and thereby having a normalized probability distribution) would not give us statistical significance values. As the reviewer correctly notes, E-values and p-values also depend on target database size. For future work, we will need to estimate Z along with E- and p-values empirically.

In the "Importance sampling alignment algorithm" subsection, we made revisions to clarify the difference between these two concepts.

(11) I agree with the following statement, but I think it needs more support. Not necessarily objective data, but something like a hypothetical scenario, or some kind of an argument illustrating the intuition. "However, when trying to improve the sensitivity of homology search, even small increases in signal are potentially useful."

For more intuition, we have added a sentence at the end of this paragraph, explaining that (for example) a 2-bit increase in homology signal would lead to a 4-fold improvement in statistical significance (E-value).

(12.) In Fig. 4B, the black line is an HPM with e_ij set to zero. Isn't this equivalent to a pHMM? Why does it perform so much better than HMMER in this test?

In the "HPMs perform promisingly" section, we now describe the difference between a pHMM and a no e_{kl} HPM. Though both models only consider primary sequence conservation, they differ in terms of training, handling of deletions, and alignment algorithm.

(13.) I suspect a bit more analysis of Fig. 4 would be helpful for some readers, and to some extent for me. Mainly, it's apparent that Infernal (Fig 4D) allows for a significant probability for G-U base pairs in comparison to the training MSA (Fig 4B). This is presumably the result of priors that lead Infernal to anticipate the Watson-Crick-compatible G-U pair. Thus, Infernal is distorting the input distribution, but in a desired way. However, I myself am a bit puzzled as to why it doesn't also allow for U-G base pairs. At any rate, the G-A and A-C pairs that the HPM allows (Fig 4C) are not desired. I think a brief explanation of these issues in Fig. 4 would help.

Infernal is taking primary sequence into account here. U is much more frequent than G at site 52 in the training MSA, while the reverse is true at site 62. Therefore, Infernal assigns a higher probability to a UG base pair than a GU base pair. We have clarified this result in the caption for Fig 5.

(14.) Minor grammar issues / typos:
"the the training alignment"
"with performance is closer to Infernal's"
"Besides improved parameterization, there are other issues to address to make HPMs as the basis for useful homology search and alignment software tools." : delete "as"
"grand number n/a" --> "grant number N/A"

Thank you, we corrected these errors.

Reviewer #2:
Reviewer #2 focuses on the reproducibility of the data given the code provided in supplement S1 Code. He was able to recreate data from the Twister ribozyme benchmark using the bash scripts in S1 Code, but was unable to reproduce the other benchmark results with the provided code.

> (1) No documentation is provided describing how to use these tools to reproduce other results presented in the manuscript. The computational requirements of performing these simulations make it difficult to repeat them, having the well documented benchmark problem provides confidence that with further documentation all simulation results presented in this manuscript could be reproduced.

We added instructions for the reproduction of the tRNA and SAM riboswitch benchmarks in S1 Code.

In addition, we have included instructions and bash scripts for the reproduction of the simulated positive control experiments that are suggested by Reviewer #3.

Reviewer #3:
Reviewer #3 considers the problem addressed by the paper to be "central to computational biology." He is "generally very positive about the manuscript," stating, "The new method is intriguing and thought provoking." The reviewer asked us to perform experiments with simulated data to better characterize "the approach under the model." In addition, the reviewer had helpful suggestions for how to improve the supplemental code.

> (1) Given the novelty of the approach and implementation, I would expect some validation using simulated data under the model, to convince that the approach works as expected. For instance, I would think that the following experiments would be informative. e.g.
>   a. Parameter estimation: generate sequences under models with specific parameters and demonstrate that the parameters can be estimated correctly from the data.
>   b. Alignment accuracy: can the method recover the correct alignment (i.e. residue-level homology) among a bunch of sequences generated by a model?
>   c. Convergence of Monte Carlo sampling: how long is sampling needed to obtain satisfactory results in the above experiments?

We have performed the experiments suggested by the reviewer, and they add to our intuition for how our model performs. We have added a brief subsection, "Characterizing HPM performance with simulated data," and a new figure, Fig 7, to the Results section describing these experiments and their results.

When training an HPM on simulated sequences generated from an HPM, we find our method is not fully able to recreate model parameters. However, we find that an HPM can accurately align the simulated sequences

on which it was trained. We also find that our Monte Carlo samples converge by 5000 iterations, after which we accept sequences.

> (2) We use synthetic sequences in order to avoid penalizing a method that identifies remote, previously unknown evolutionary relationships. Decoys are created with characters drawn i.i.d from the nucleotide composition of the positive test sequences, with the length of each decoy matching a randomly-selected positive test sequence." One risk with this approach is that the generated sequences have properties that are pretty different from real sequences (i.i.d.). I feel this should be at least discussed. But two simple strategies which could be of interest would be to invert the sequences (which keeps local composition) or shuffling the sequence in local windows.

We use decoys with characters drawn i.i.d. for the sake of simplicity. We made revisions In the Discussion to acknowledge that there are methods for generating decoys that more closely resemble real biological sequences, stating that further HPM benchmarking will use shuffled decoys.

> (3) I would expect the "no e_{kl} HPM" model to performs similarly or worse than pHMM. Yet it gave better performance on the tRNA model. Any sense why this could be the case?

In the "HPMs perform promisingly" section, we now describe the difference between a pHMM and a no e_{kl} HPM. Though both models only consider primary sequence conservation, they differ in terms of training, handling of deletions, and alignment algorithm.

> (4) "In the three alignment benchmarks (see table 2), the all-by-all HPM is more accurate than the no e_{kl} HPM. We conclude e_{kl}'s generally add sensitivity to remote homology search and alignment." Were parameters also fitted with the constraint that e_{kl} = 0?

Yes, the no e_{kl} HPMs are trained using pseudolikelihood maximization with all e_{kl} terms constrained to 0 throughout training. We have added this explanation to the "HPMs perform promisingly" section.

> (5) Potts model fitting need very large MSAs. This is likely to be the case here. It would be interesting to see if the number of sequences used for training has an impact on the performance. But in any case, this could be discussed.

We agree that studying the effect of training alignment depth on HPM performance in remote homology search is necessary if HPMs are to become a state-of-the-art tool. In the Discussion, we say this is a topic for future work. In addition, we reference a paper that studies how training set size affects a Potts model's accuracy in structure and fitness prediction in protein.

> (6) Code is provided, but this is quite some way to being "usable". There is hardly any documentation, and no test. The code has some external dependencies which makes compiling non-

> trivial. No binary is provided. I understand the authors regard their contribution more of a proof of concept than a tool (and indeed the senior author has an outstanding track record providing usable tools) but a little effort toward making the code more usable would surely encourage reuse and extensions. (Actually, I later found that the code and readme files provided as supplementary materials provides sample files and compiling instructions—please include these on the GitHub repo as well please, as most readers are likely to retrieve the code from Github rather than a supplementary file to the manuscript).

We have added readme files and additional documentation to a new GitHub repository

We have also added bash scripts to automatically clone and compile the external dependencies (HMMER, Infernal, and the Easel sequence library) from the appropriate repositories.

We are unable to provide HMMER, Infernal, and Easel directly with S1 Code because of limitations on the size of supplementary files. Instead, we provide the aforementioned bash scripts with the supplement.

Again, we thank you and the reviewers for your comments, and we hope our revised manuscript is viewed favorably!

Sincerely,

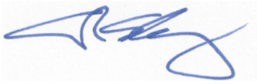

Sean R. Eddy
